# Supplementary figures and images for: BehaveAI enables rapid detection and classification of objects and behavior from motion
Source: PLoS Biol. 2026 Feb 20;24(2):e3003632. doi: 10.1371/journal.pbio.3003632 (PMC12922998; doi:10.1371/journal.pbio.3003632)

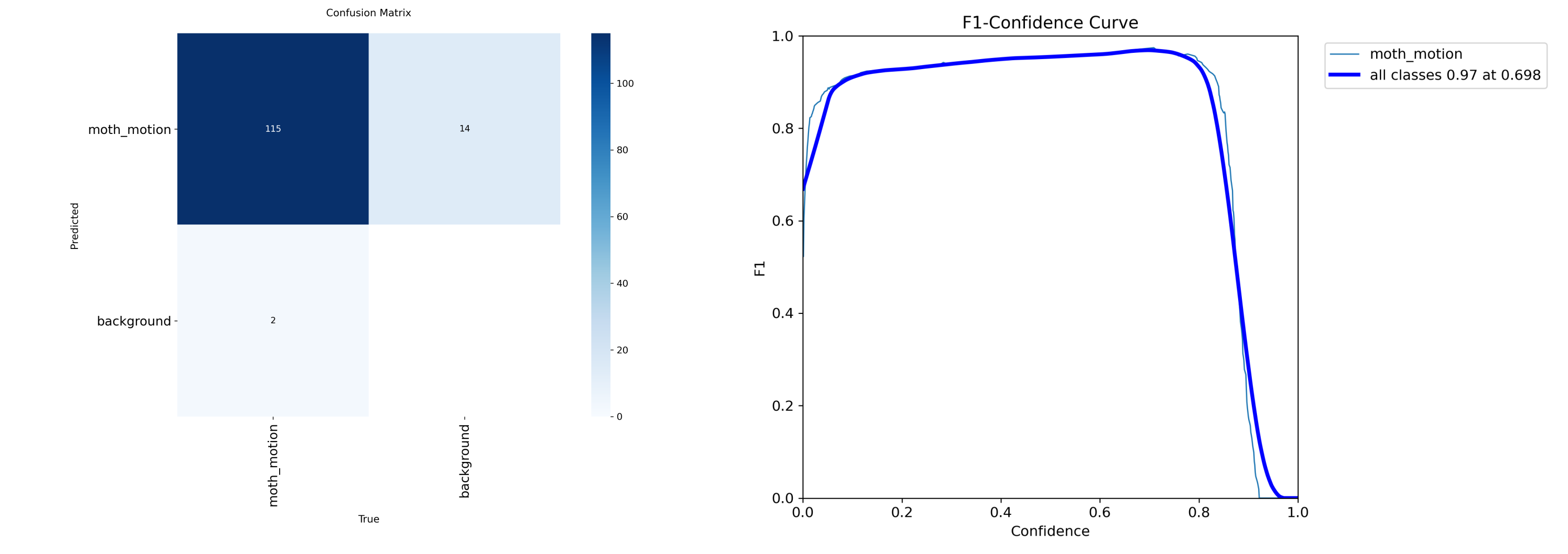

Supplement: S1 Fig — The data underlying this Figure are available here: https://doi.org/10.6084/m9.figshare.30531116. (TIFF) [file pbio.3003632.s001.tiff]

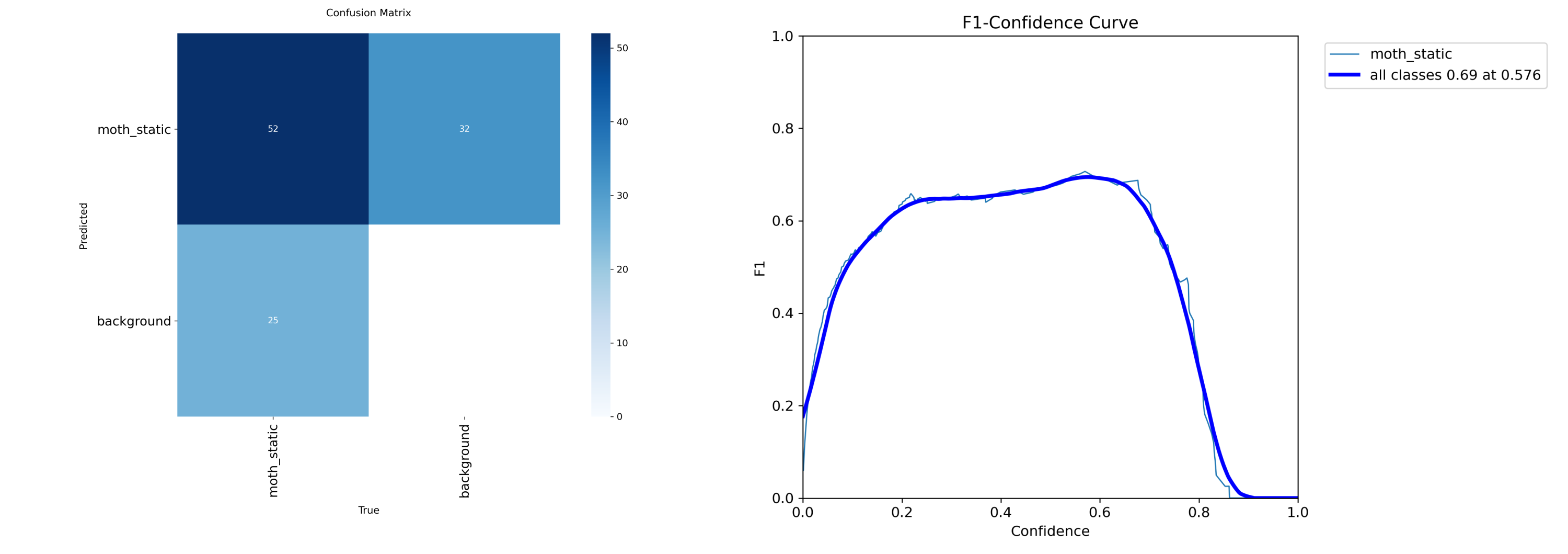

Supplement: S2 Fig — The data underlying this Figure are available here: https://doi.org/10.6084/m9.figshare.30531116. (TIFF) [file pbio.3003632.s002.tiff]

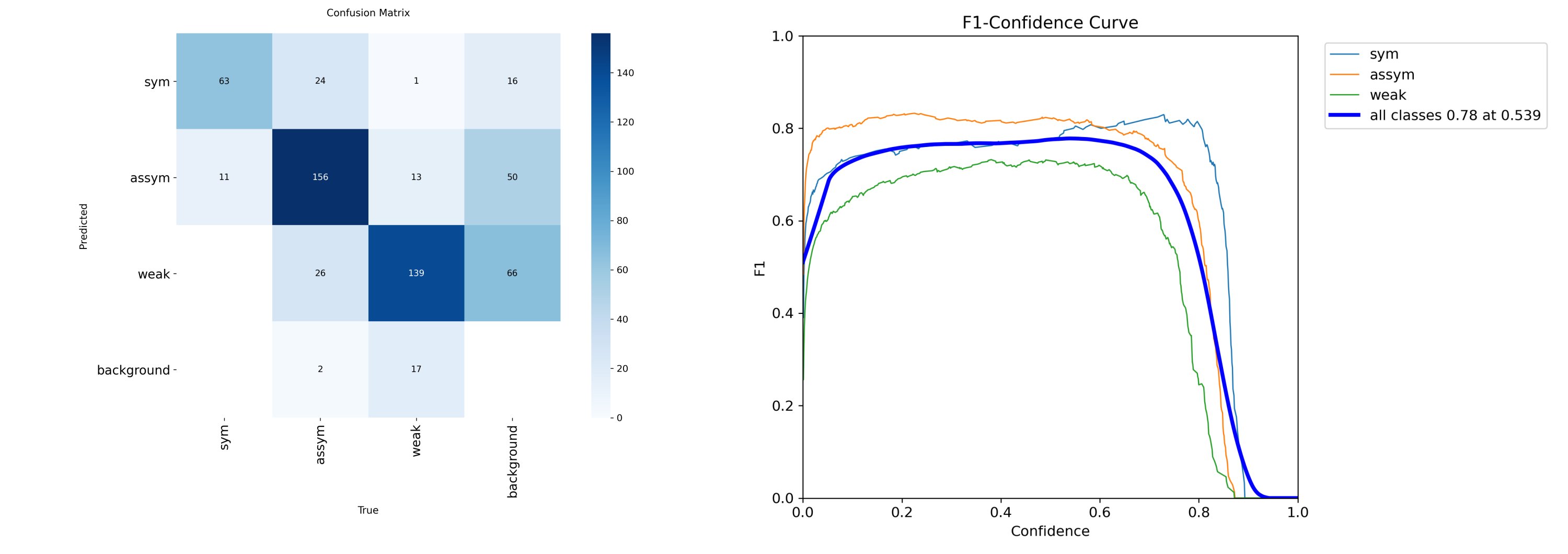

Supplement: S3 Fig — The data underlying this Figure are available here: https://doi.org/10.6084/m9.figshare.30531116. (TIFF) [file pbio.3003632.s003.tiff]

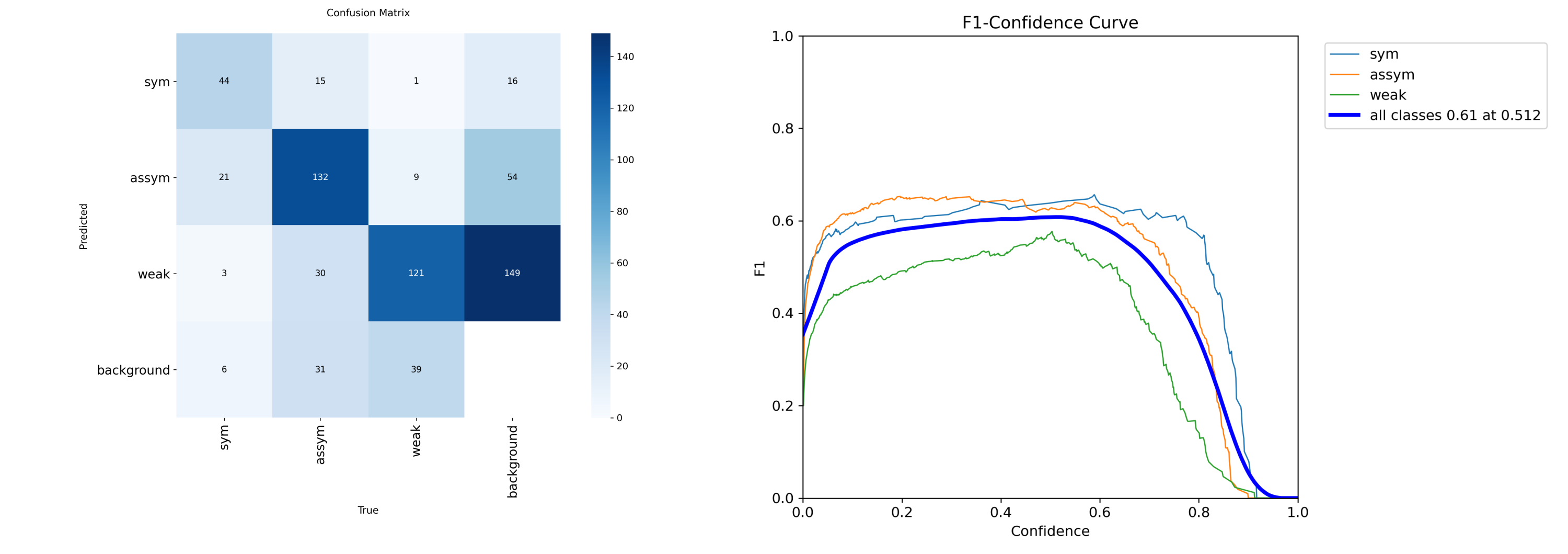

Supplement: S4 Fig — The data underlying this Figure are available here: https://doi.org/10.6084/m9.figshare.30531116. (TIFF) [file pbio.3003632.s004.tiff]

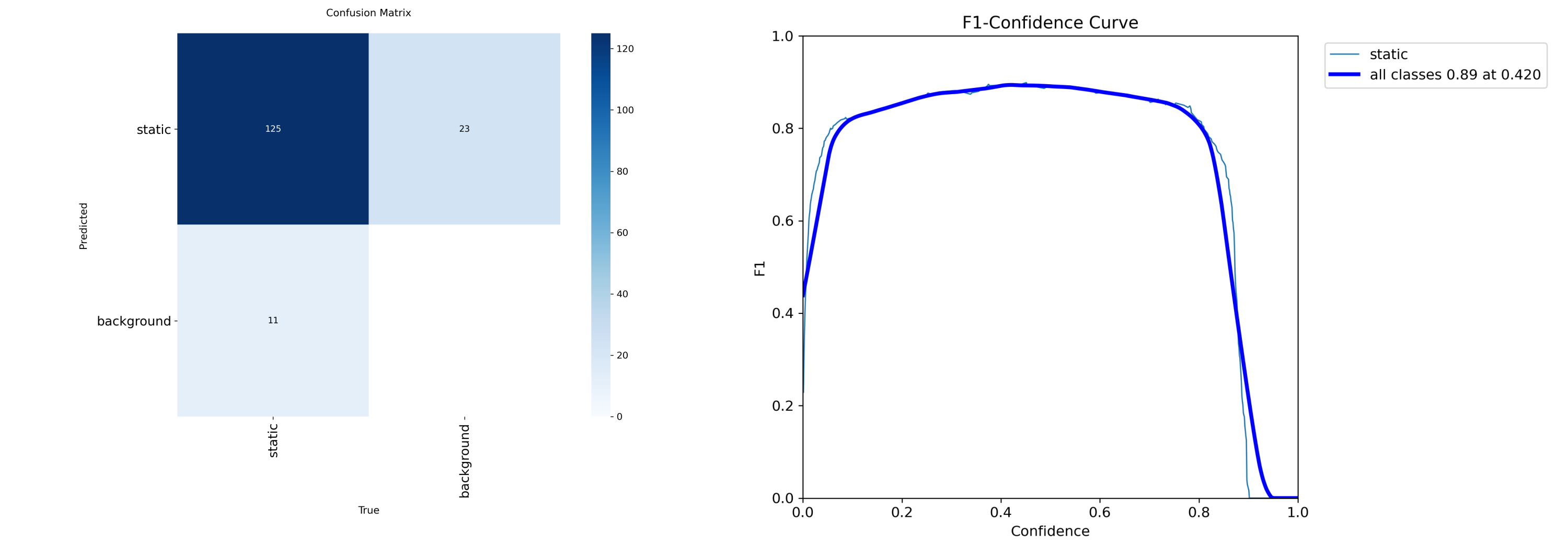

Supplement: S5 Fig — The data underlying this Figure are available here: https://doi.org/10.6084/m9.figshare.30531116. (TIFF) [file pbio.3003632.s005.tiff]

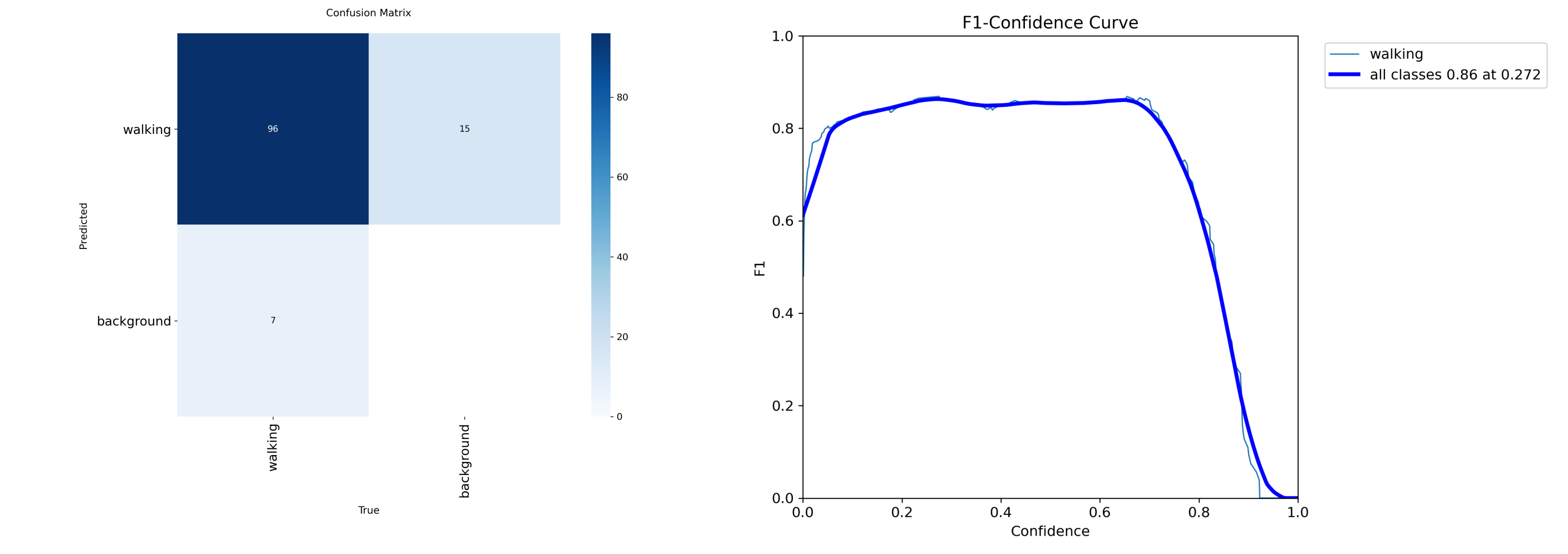

Supplement: S6 Fig — The data underlying this Figure are available here: https://doi.org/10.6084/m9.figshare.30531116. (TIFF) [file pbio.3003632.s006.tiff]

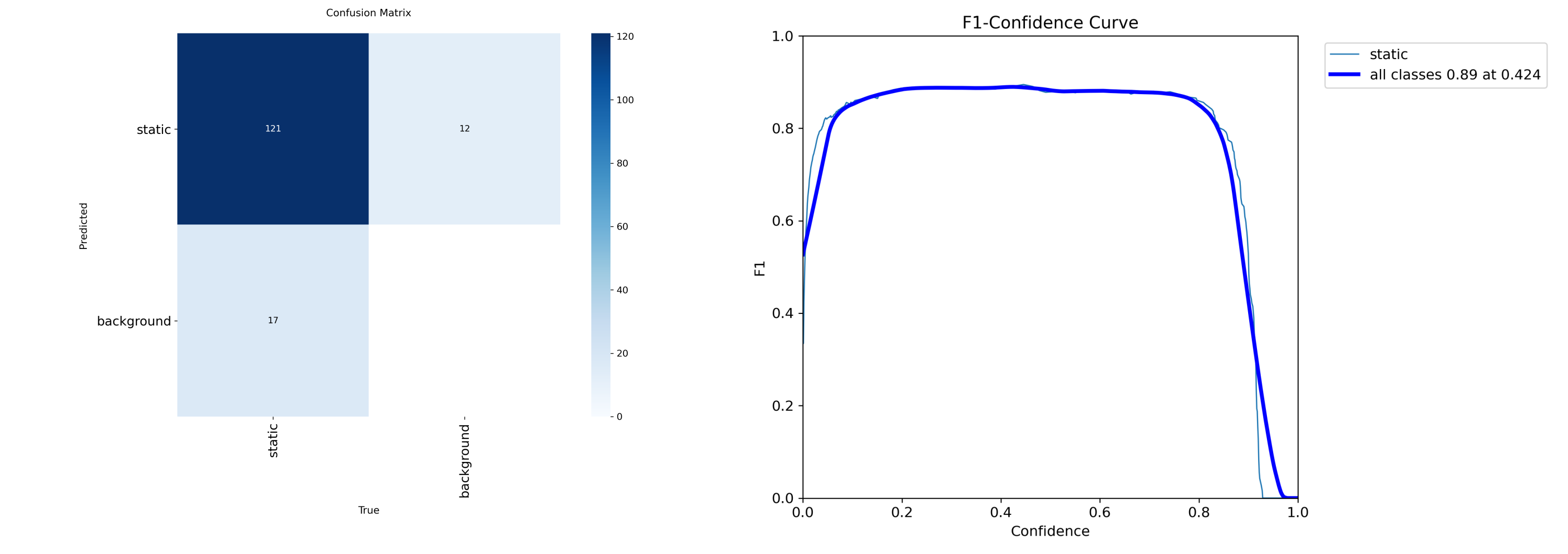

Supplement: S7 Fig — The data underlying this Figure are available here: https://doi.org/10.6084/m9.figshare.30531116. (TIFF) [file pbio.3003632.s007.tiff]

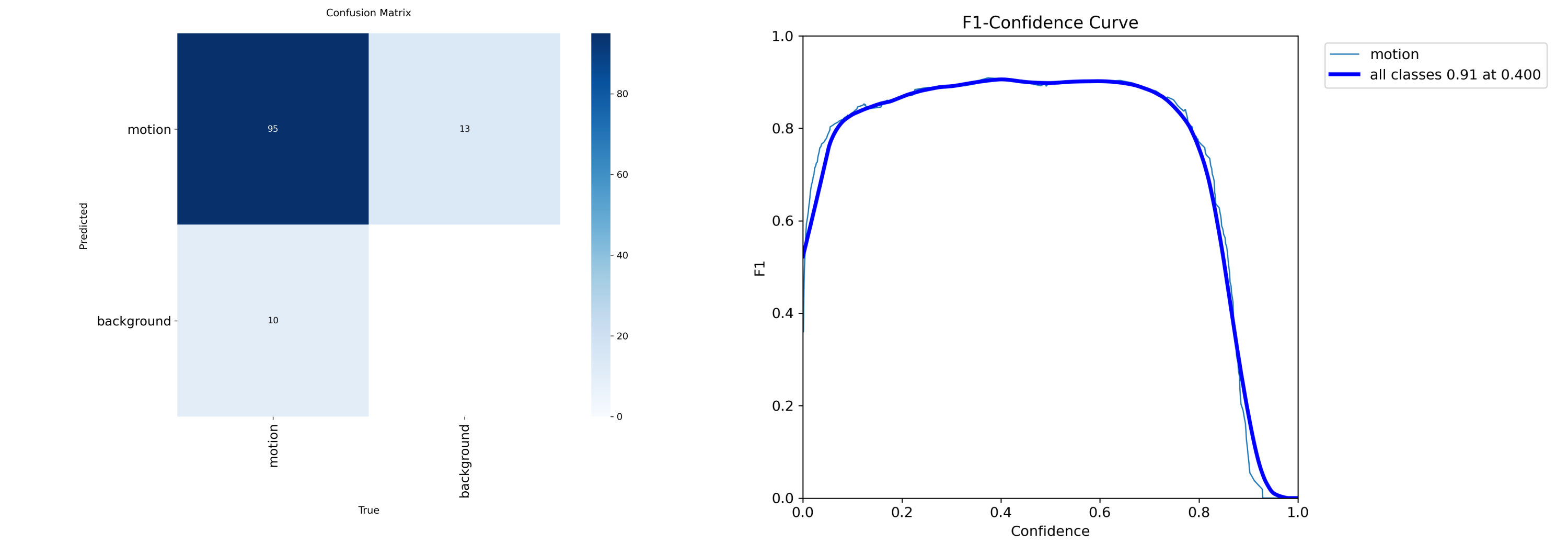

Supplement: S8 Fig — The data underlying this Figure are available here: https://doi.org/10.6084/m9.figshare.30531116. (TIFF) [file pbio.3003632.s008.tiff]

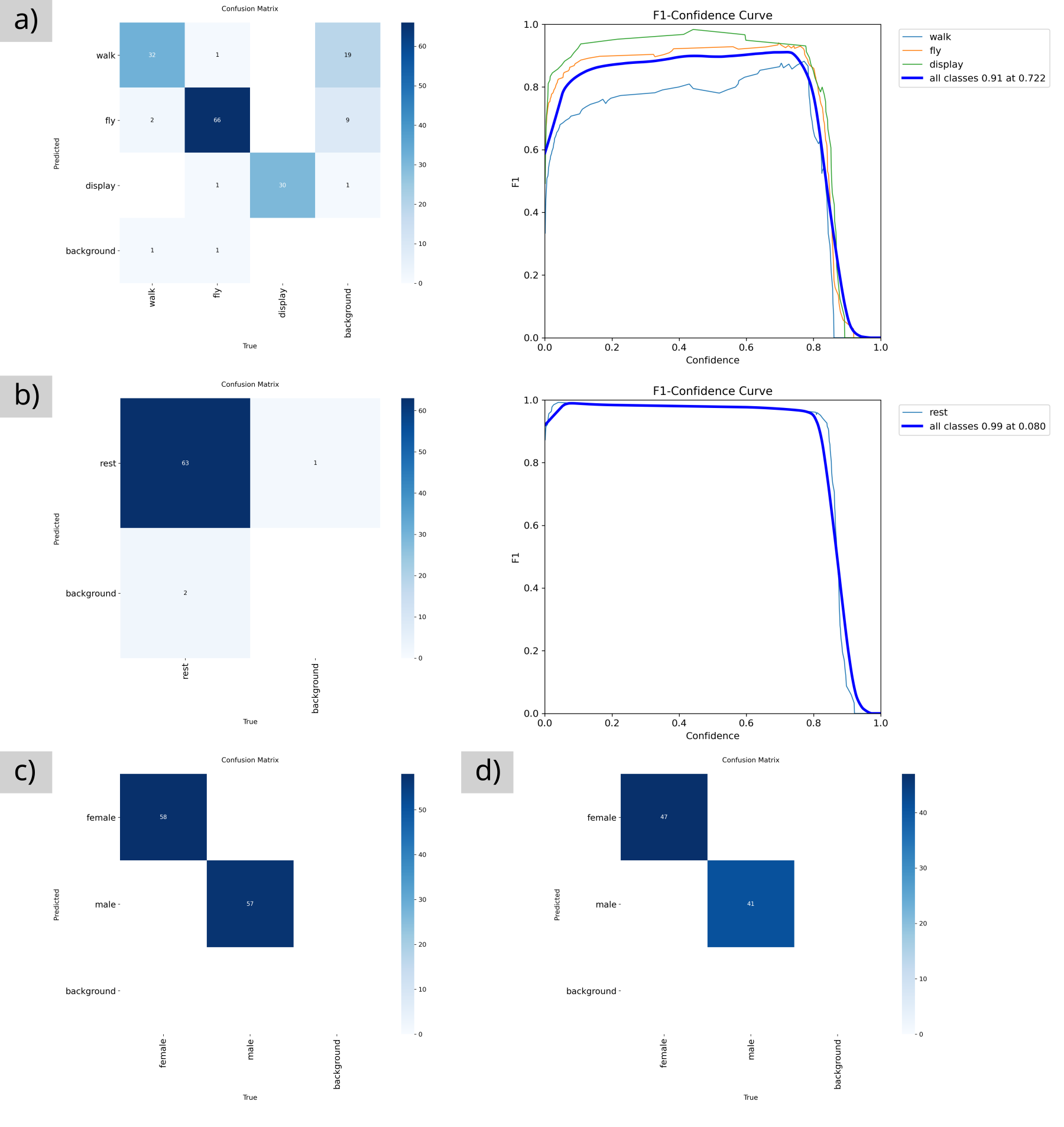

Supplement: S9 Fig — a) primary motion model, (b) primary static model, (c) secondary model determining sex while flies are at rest, and d) while flies are walking. The data underlying this Figure are available here: https://doi.org/10.6084/m9.figshare.30531116. (TIFF) [file pbio.3003632.s009.tiff]
