# Supplementary material for: BehaveAI enables rapid detection and classification of objects and behavior from motion
Source: PLoS Biol. 2026 Feb 20;24(2):e3003632. doi: 10.1371/journal.pbio.3003632 (PMC12922998; doi:10.1371/journal.pbio.3003632)
Supplement: Attachment — Submitted filename: BehaveAI_reviewer responses.pdf [file pbio.3003632.s012.pdf]

Thank you for your patience while your manuscript "BehaveAI: a framework for rapidly detecting and classifying objects and behaviour from motion" was peer-reviewed at PLOS Biology. It has now been evaluated by the PLOS Biology editors, an Academic Editor with relevant expertise, and by two independent reviewers.

Based on the reviews, which you will see are highly favourable, we are likely to accept this manuscript for publication, provided you satisfactorily address the remaining points raised by the reviewers and the following data and other policy-related requests.

IMPORTANT - please attend to the following:

a) We try to avoid punctuation in Titles. Please could you change your Title to avoid this? (for example, you could simply start it with "BehaveAI is a framework for…")

We propose changing the title to 'BehaveAI enables rapid detection and classification of objects and behaviour from motion'

b) Please address the comments from the reviewers.

Done.

c) Please address my Data Policy requests below; specifically, we need you to supply the numerical values underlying Figs 3B, S1-S9, either as a supplementary data file or as a permanent DOI'd deposition.

These data are all available via the Figshare DOI.

d) Please cite the location of the data clearly in all relevant main and supplementary Figure legends, e.g. "The data underlying this Figure can be found in S1 Data" or "The data underlying this Figure can be found in https://zenodo.org/records/XXXXXXXX

Done.

e) I note that your code is in GitHub (https://github.com/troscianko/BehaveAI); many thanks for providing this. However, because Github depositions can be readily changed or deleted, please also make a permanent DOI'd copy (e.g. in Zenodo) and provide this URL.

Of course. We have made a number of snapshots with DOIs in Zenodo as we have iterated the versions. We will include the latest version as a zenodo DOI.

As you address these items, please take this last chance to review your reference list to ensure that it is complete and correct. If you have cited papers that have been retracted, please include the rationale for doing so in the manuscript text, or remove these references and replace them with relevant current references. Any changes to the reference list should be mentioned in the cover letter that accompanies your revised manuscript.

In addition to these revisions, you may need to complete some formatting changes, which you will receive in a follow up email. A member of our team will be in touch with a set of requests shortly. If you do not receive a separate email within a few days, please assume that checks have been completed, and no additional changes are required.

We expect to receive your revised manuscript within four weeks.

To submit your revision, please go to https://www.editorialmanager.com/pbiology/ and log in as an Author. Click the link labelled 'Submissions Needing Revision' to find your submission record. Your revised submission must include the following:

- a cover letter that should detail your responses to any editorial requests, if applicable, and whether changes have been made to the reference list
- a Response to Reviewers file that provides a detailed response to the reviewers' comments (if applicable, if not applicable please do not delete your existing 'Response to Reviewers' file.)
- a track-changes file indicating any changes that you have made to the manuscript.

NOTE: If Supporting Information files are included with your article, note that these are not copyedited and will be published as they are submitted. Please ensure that these files are legible and of high quality (at least 300 dpi) in an easily accessible file format. For this reason, please be aware that any references listed in an SI file will not be indexed. For more information, see our Supporting Information guidelines:
https://journals.plos.org/plosbiology/s/supporting-information

***Published Peer Review History***
Please note that you may have the opportunity to make the peer review history publicly available. The record will include editor decision letters (with reviews) and your responses to reviewer comments. If eligible, we will contact you to opt in or out. Please see here for more details:
https://plos.org/published-peer-review-history/

***Press***
Should you, your institution's press office or the journal office choose to press release your paper, please ensure you have opted out of Early Article Posting on the submission form. We ask that you notify us as soon as possible if you or your institution is planning to press release the article.

***Protocols deposition***
To enhance the reproducibility of your results, we recommend that if applicable you deposit your laboratory protocols in protocols.io, where a protocol can be assigned its own identifier (DOI) such that it can be cited independently in the future. Additionally, PLOS ONE offers an option for publishing peer-reviewed Lab Protocol articles, which describe protocols hosted on protocols.io. Read more information on sharing protocols at https://plos.org/protocols?utm_medium=editorial-email&utm_source=authorletters&utm_campaign=protocols

Please do not hesitate to contact me should you have any questions.

Sincerely,

Roli

Roland Roberts, PhD
Senior Editor
rroberts@plos.org
PLOS Biology

------------------------------------------------------------------------

DATA POLICY:

You may be aware of the PLOS Data Policy, which requires that all data be made available without restriction: http://journals.plos.org/plosbiology/s/data-availability. For more information, please also see this editorial: http://dx.doi.org/10.1371/journal.pbio.1001797

Note that we do not require all raw data. Rather, we ask that all individual quantitative observations that underlie the data summarized in the figures and results of your paper be made available in one of the following forms:

1) Supplementary files (e.g., excel). Please ensure that all data files are uploaded as 'Supporting Information' and are invariably referred to (in the manuscript, figure legends, and the Description field when uploading your files) using the following format verbatim: S1 Data, S2 Data, etc. Multiple panels of a single or even several figures can be included as multiple sheets in one excel file that is saved using exactly the following convention: S1_Data.xlsx (using an underscore).

2) Deposition in a publicly available repository. Please also provide the accession code or a reviewer link so that we may view your data before publication.

Regardless of the method selected, please ensure that you provide the individual numerical values that underlie the summary data displayed in the following figure panels as they are essential for readers to assess your analysis and to reproduce it: Figs 3B, S1-S9. NOTE: the numerical data provided should include all replicates AND the way in which the plotted mean and errors were derived (it should not present only the mean/average values).

IMPORTANT: Please also ensure that figure legends in your manuscript include information on where the underlying data can be found, and ensure your supplemental data file/s has a legend.

Please ensure that your Data Statement in the submission system accurately describes where your data can be found.

------------------------------------------------------------------------

CODE POLICY

Per journal policy, if you have generated any custom code during the course of this investigation, please make it available without restrictions. Please ensure that the code is sufficiently well documented and reusable, and that your Data Statement in the Editorial Manager submission system accurately describes where your code can be found.

Please note that we cannot accept sole deposition of code in GitHub, as this could be changed after publication. However, you can archive this version of your publicly available GitHub code to Zenodo. Once you do this, it will generate a DOI number, which you will need to provide in the Data Accessibility Statement (you are welcome to also provide the GitHub access information). See the process for doing this here:
https://docs.github.com/en/repositories/archiving-a-github-repository/referencing-and-citing-content

------------------------------------------------------------------------

DATA NOT SHOWN?

- Please note that per journal policy, we do not allow the mention of "data not shown", "personal communication", "manuscript in preparation" or other references to data that is not publicly available or contained within this manuscript. Please either remove mention of these data or provide figures presenting the results and the data underlying the figure(s).

------------------------------------------------------------------------

REVIEWERS' COMMENTS:

Reviewer #1:
[identifies himself as Ammon Perkes]

Reviewing this manuscript was delightful. I want to say, up front, I think this is really excellent work. I have several suggestions below that I hope you will consider and apply, but at the risk of being called a soft reviewer, I think this already merits publication in PLOS Bio as is. I have used many different tools for tracking animal behavior and written plenty of code to do it myself, and this is by far the most effective and useful tool I have ever worked with. It reminded me a lot of the original idTracker paper, where they managed to do something seemingly miraculous just with clever feature selection. Anyway, that is probably enough effusive praise for now, but I am very excited about this tool and am very appreciative of all of the work and ingenuity that went into making it.

This is a heartening appraisal, that is also backed up with thorough testing and thought given the detail below. We are hugely grateful for all the time and effort that has gone into testing.

I've divided my review into comments about the manuscript and comments about the package itself, the latter of which is maybe slightly outside the scope of the review, but I think it could be helpful.

Concerning the manuscript:

I found the manuscript very easy to read and extremely clear. It directs people up front to the code and documentation, which is very helpful. I only have a few minor comments.

- I think the static vs motion comparisons are useful, and I agree that you don't need to do detailed performance comparisons with existing models on all these datasets. That said, I think it would be beneficial, where possible, to mention as context what the state of the field is for performance. For example, with the POP-3D dataset, you mention that your results are comparable to Chan et al, but unless I missed it, I don't think you ever state what that performance is. I think this is useful information for readers to know, with the existing caveat that this of course isn't necessarily a 1:1 comparison.

Good point - we have added the equivalent results from Chan et al 2025 under the Pigeon behaviour – 3D-POP dataset case study. We have added the following:

“Chan et al. 2025 report a precision and recall of 0.77 and 0.70 respectively with this dataset, although note that the behaviours are slightly differently structured.”

- Line 209 is missing a period and there's a "LINK" placeholder on line 248. Obviously very minor stuff, but make sure to get a careful readthrough during copy editing.

Fixed – link to the Github page added.

- As written, line 223 feels a bit perfunctory. I don't think you even necessarily even need to include it, but if you do, I think you could expand it slightly. I think your colour-from-motion approach is more innovative and potentially impactful than you give yourself credit, and I think it could turn out to be really powerful for a range of computer vision tasks, particularly in low-powered robotics applications.

We have rewritten this sentence to emphasise the potential utility of the wider strategy we adopted. The section now reads:

“The utility of the colour-from-motion strategy – or similar solutions that encode image difference over a range of different timescales – should also be explored in fields beyond the biosciences. For example, its ability to quantify acceleration and

deceleration of objects within a single frame could be valuable in machine vision tasks such as robotic applications or autonomous vehicle control."

Concerning the method, does it work as promised?

In short, yes, absolutely. I was skeptical when reading the abstract that you could really deliver what you were promising, but just in the week of playing around with it, I've been shocked at how effective it is. With around 25 frames, I was able to track a difficult dataset (baby fish swimming over gravel), where before I had used this complex combination of SLEAP trained on several hundred frames and MOG-Backround subtraction. And I was able to train and run it on my personal laptop, all in less than an hour, at 20fps. On GPUs, the speed gains are obviously dramatic, and there are two huge pipelines that I'm already in the process of moving over from SLEAP to BehaveAI because of the improved speed and accuracy that we're getting. I haven't tested the secondary classifiers as much, but already, I think this has the potential to become a mainstay of the biology world, and probably other contexts in fields I haven't even considered. It's obvious that (compared to a lot of existing packages that were designed with neuroscience or collective behavior in mind) this was really built by and for people interesting in quantifying animal behavior in a naturalistic context.

We are absolutely delighted that you tested our framework so thoroughly, and that it's working well for you.

Is it sufficiently easy to use to be useful?

I found it quite easy to install based on the documentation, and a CS undergrad I asked to try it did as well. Already, this is a lot easier to use than most of the packages out there. I do have a few suggestions, but these are more like feature requests than reviewer comments, so I don't think fixing this needs to happen prior to publication. That said, I think some improvements could make this a lot more usable to the bulk of biologists, who might be less comfortable with working in the command line.

Many thanks for these detailed suggestions, as you'll see below we've taken many of the key suggestions on board and have made some major changes to increase usability.

Significantly, we have overhauled the framework so that it uses a single install, and the user can create 'projects', which are hopefully more intuitive. Users then have a settings graphical user interface (GUI) through which they can specify their model structures, and parameter settings. This system makes it more difficult for users to inadvertently make errors.

We have also integrated the 'live' tool with this system, allowing any user with a webcam to use the BehaveAI user interface for the complete process, i.e. video recording, annotation, model training, and live-tracking with inference or batch processing. We have also improved edge (real-time, live processing) performance, achieving 11FPS at 640x480 px for a hierarchical model on a Raspbbery Pi 5, and >30FPS at 1280x720 px on a standard laptop without graphics acceleration.

The extent of these substantial user experience changes means that our existing introduction and user-guide videos are now out-of-date. Rather than confuse readers with old user interfaces and workflows we have removed the links to the videos. If time permits we may be able to create new videos before the proof stage, and will include them with the final document if possible.

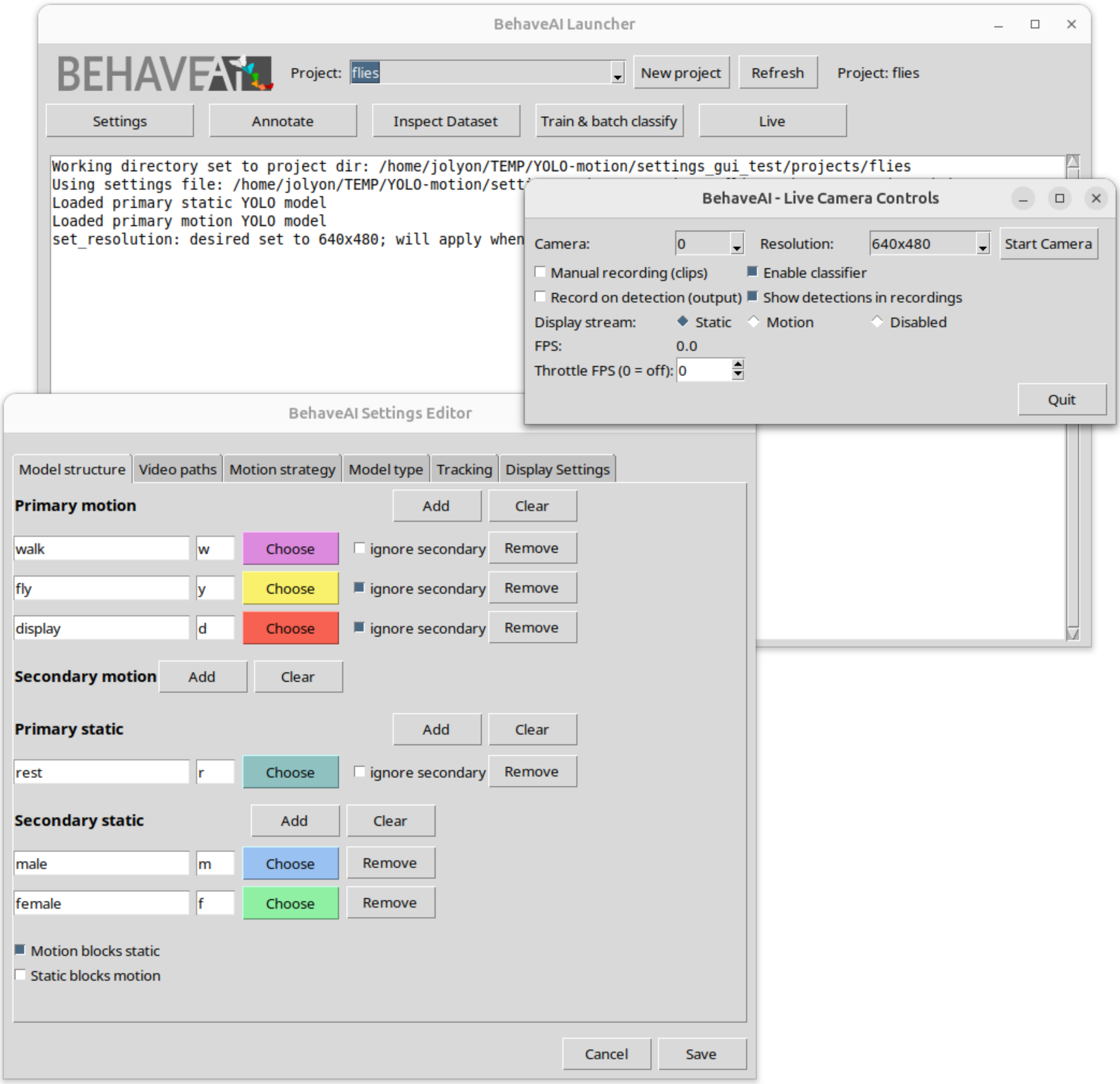


- I think the idea that each project essentially having a separate instantiation for the code is a little counter-intuitive for our app-centric world. I would suggest making this mindset slightly more clear in the documentation from the outset. My undergrad ran into trouble with some mismatches between some initial annotations and a later download of your dataset, causing batch processing to fail, which I think probably stems from not making a fresh directory.

Agreed. The updated framework now doesn't require this. The GUI automatically handles separate 'projects', and the user can manually specify directory paths for training clips, and the input/output batch processing directories.

- I think editing settings files is a pretty big barrier to entry for a lot of people, and I would really encourage you make it possible to do this from the GUI. This would also help prevent bugs caused from incorrect editing of the settings file. SLEAP for example does this really well, and is a big part of why that platform is so useful.

Likewise, we agree, and have bound this up into a settings GUI (see screenshot above). Hopefully this will substantially reduce barriers to entry and user errors e.g. hierarchical model structures can become quite complex, with plenty of opportunity for user error.

The new GUI also warns users if they attempt to change the model structure after starting the annotation datasets as this is likely to invalidate an existing database.

- I think having a written guide walking users through an example dataset with step-by-step instructions would be really helpful. The documentation almost has this already, and the video guide does this really well, but I think an explicit "label these 20 frames, now press escape, now click train & batch classify" would help get users going quickly.

Agreed, we have created a project wiki on Github that has more detailed written user instructions, with a clear structure.

- A very helpful feature would be a way to see, in the annotator, which frames have already been labeled.

This would indeed be useful. We have now added markers to the tracking bar to indicate which frames have been labelled, and shortcuts to skip forwards or backwards to the next or previous frame containing annotations. More broadly, we have modified the annotation interface to automatically load and visualise all previous annotations, allowing users to assess and edit these in real-time, while providing an improved overview of the dataset. This update is currently undergoing testing, but we hope to release it in the coming days.

- On my laptop (a macbook in darkmode) the terminal readout in the GUI is darkgrey-on-black and basically invisible. It looked great on my ubuntu desktop.

We'll tag this for future updates (we don't have easy access to MacOS). A user option for background colour would be straightforward.

- When doing the second round of annotations, a visualization showing which frames had annotations would make it easier to see how well it worked and also help locate frames where 0 animals were detected.

Yes, this could be useful in some cases, though hopefully users with lots of videos can simply be systematic in their sampling. This relates to the above request too.

- I think it could be more clear in the documentation that you need to set all the parameters in the settings file prior to running the code and starting annotations. You mention that towards the end of the documentation, but it would be helpful for that to be made clear from the start.

Hopefully the new GUI-based settings system solves this issue.

- The way the zoomed-in box in the annotation GUI distorts around the edges is a bit strange. I assume this is to prevent labeling outside the frame, but I wonder if there is another approach that would not distort the image.

We can test out alternatives – just cropping would make sense – and flag for future updates.

- There are a few typos in the documentation ("determin" and "insepct", probably others)

Fixed.

I'm sure there are unlimited improvements you could make to this, many of which you have already considered. You already mention ideas you have for active development, and I would strongly encourage you, if you haven't already, to plan for dedicated developers whose primarily role is to improve this package and handle bugs and help requests as they arise, rather than it all falling to you to find the time in between all your other responsibilities. There are so many packages that were published but aren't actively maintained that quickly become unusable. I really do think this has the potential to be the most used tracking

platform for animal behavior, but only if you can address people's challenges as they come up and continue to make it more useful over time.

This will indeed be a major challenge! By releasing the project under a GNU license we hope that – should it prove valuable – others would simply be able to 'fork' (copy) and maintain another version in Github or elsewhere. We also aim to keep the central pipeline as simple and streamlined as possible for rapid integration with new YOLO releases and minimum maintenance requirements.

Overall, I think you have created exactly what it says in the title, and I think this will be a game changer for everyone studying animal behavior, making a great deal of tracking and behavioral classification that was previously difficult or impossible fast and straightforward. I look forward to seeing all the cool new science that takes advantage of this platform.
Many thanks

Reviewer #2:

This is a cool paper. Its a bit out of my area of expertise, but I appreciate that the color-for-motion idea is very clever and looks like a major advance for using AI to study animal behavior from videos. This is an important topic of broad interest and looks like a good advance. Sorry I'm not qualified to comment on the technical aspects.

Many thanks for the appraisal.

Introduction had a nice first few paragraphs that do a good job at reviewing the field.

The fourth intro paragraph needs some work, isn't well connected with the earlier mateiral, work on transition sentences and conceptual flow especially at the start.

Agreed – we have altered the text to lead on from the prior paragraph more coherently.

The final intro paragraph is also a little confusing, as it starts by stating objectives but then starts talking about benchmarking and kind of trails off.

We agree that this could be improved. We have moved the detail regarding benchmarking to the methods section, and added a final sentence that draws the paragraph to a close more clearly. Many readers might expect benchmarking to be performed, however as noted by reviewer 1, this has been problematic in the field, which we feel needs to be pointed out.

Figure 2 - what do the colors of the bottom frames indicate?

These were simply coded to show the progression of time. We have altered these to reduce any confusion – they are all now the same grey level.
